# Wildfire smoke and pediatric asthma control in the Northeastern United States: a cross-sectional study

**DOI:** 10.1186/s12940-025-01245-9

**Published:** 2025-12-11

**Authors:** Anna K. Maassel, Paige Brochu, Valerie S. Harder, Taylor H. Ricketts, Stephen J. Teach, Keith J. Robinson

**Affiliations:** 1 Rubenstein School of Environment and Natural Resources, University of Vermont, 81 Carrigan Drive, Burlington, VT 05405 USA; 2 Gund Institute for Environment, University of Vermont, 210 Colchester Avenue, Burlington, VT 05405 USA; 3 Vermont Child Health Improvement Program, University of Vermont Larner College of Medicine, 1 South Prospect Street, Burlington, VT 05401 USA; 4 Department of Pediatrics, University of Vermont Larner College of Medicine, 89 Beaumont Avenue, Burlington, VT 05405 USA; 5 Department of Emergency Medicine, University of Vermont Larner College of Medicine, 89 Beaumont Avenue, Burlington, VT 05405 USA; 6 Emergency Department, University of Vermont Medical Center, 111 Colchester Avenue, Burlington, VT 05401 USA; 7 Pediatric Pulmonology, University of Vermont Children’s Hospital, 111 Colchester Avenue, Burlington, VT 05401 USA

**Keywords:** Asthma, Pediatrics, Wildfire, Particulate matter, PM_2.5_, Planetary health, Climate change, Asthma control

## Abstract

**Background:**

Poor air quality due to smoke from distant wildfires is a growing risk for the Northeastern United States, a region largely unaffected by these events until recently. Despite this emerging threat, few studies have examined the effect of wildfire smoke on respiratory health in this region. We investigated the association between wildfire smoke exposure and pediatric asthma control in Vermont and upstate New York.

**Methods:**

We extracted data from the electronic health records of youth aged 3–21 years diagnosed with asthma within a single regional healthcare system and included three clinical measures of asthma control: Test for Respiratory and Asthma Control in Kids (3–4 years), Asthma Therapy Assessment Questionnaire (5–21 years), and the National Heart, Lung and Blood Institute (NHLBI) asthma control guidelines (3–21 years). We first compared asthma control in the smoke-affected summer of 2023 to the largely unaffected summers of 2022 and 2024 using regression models, controlling for pollen exposure. We then obtained airborne particulate matter (PM_2.5_) values within ZIP codes and used regression models to investigate the association between asthma control and PM_2.5_ during the smoke-affected summer of 2023.

**Results:**

The study sample included 1,217 encounters (mean age 9.1 ± 4.4 years, 57% male). Asthma control was significantly worse in the severely smoke-affected summer of 2023 versus 2022 for two of the three clinical measures but was not different between 2023 and 2024 for any of the clinical measures. Within summer 2023, there were no significant associations between ZIP code–level PM_2.5_ and asthma control for any of the three clinical measures.

**Conclusions:**

Wildfire smoke exposure in the Northeast was associated with decreased asthma control in this pediatric population, though not consistently across years and all clinical measures. As climate change drives longer and more intense wildfire seasons, continued monitoring is needed to understand the impact on pediatric respiratory health in this historically low-exposed region.

## Background

 Connections between climate change and human health are becoming increasingly clear, but research gaps still exist in predicting and quantifying those relationships. The World Health Organization estimates that climate change will cause an additional 250,000 deaths annually from 2030 to 2050 [1]. These health impacts are driven by factors such as increasing temperature [2, 3], loss of biodiversity [4], and more frequent extreme weather events [5, 6]. Together, these factors are likely to drive disease spread [7], increase hunger and malnutrition [8, 9], and exacerbate existing health conditions [10, 11].

One such relationship receiving more scientific and policy attention is between wildfire smoke and respiratory health. In the United States (US), wildfire seasons have lengthened and intensified over the past decades [12] and are predicted to increase in the future [13]. Several reviews corroborate the negative health impacts of wildfire smoke [14–18]. These impacts are greater on vulnerable populations, including children [19, 20] and those with pre-existing cardiovascular and respiratory diseases [21, 22].

Among these chronic diseases is pediatric asthma. Over four million children under 18 years in the US (6%) have asthma [23], and it disproportionately affects under-resourced and minority children and adolescents [24]. In the Northeast, childhood asthma rates are particularly concerning. Vermont has the 2nd highest rate of pediatric asthma in the country [25], making affected youth potentially vulnerable to smoke from distant wildfires. This vulnerability was tested in the summer of 2023, when severe wildfires burned in Quebec, Canada, sending dense smoke across the border into the Northeastern United States [26].

To date, most studies on the effects of wildfire smoke and respiratory health outcomes have focused on geographic areas that frequently experience wildfires, such as the western US and Australia [20]. These studies typically measure the impact of locally produced wildfire smoke and air pollution [21]. Further, these studies primarily rely on acute measures of impact, such as emergency department visits and hospitalizations [19].

This study extends and complements existing research by focusing on a geographic area becoming newly familiar with wildfire smoke (the Northeastern United States) and studying multiple measures of asthma control utilized in outpatient settings. A patient’s asthma control is a product of the severity of the patient’s asthma, medical management, self-management, and environmental exposures, and it predicts other health outcomes like health care utilization, quality of life, and functional status [27]. An asthma-related visit to the emergency department can often be avoided altogether with optimal patient management [28], so it is important to understand the impacts of wildfire smoke on patients outside of the emergency department to best inform care.

The objective of this study is to determine the relationship between exposure to airborne particulate matter from wildfire smoke and pediatric asthma control levels in Vermont and upstate New York. We first compared asthma control among years differentially affected by wildfire smoke, and we then examined asthma control during the smoke-affected summer of 2023. We hypothesized that wildfire smoke exposure (including higher PM_2.5_ levels) would both be associated with worse asthma control.

## Methods

We conducted two analyses to investigate the relationship between wildfire smoke and pediatric asthma control. First, we compared asthma control scores among the summers of 2022, 2023, and 2024, using year as a proxy for smoke exposure. We then examined the relationship between ZIP code–level PM_2.5_ and asthma control levels within the severely smoke-affected summer of 2023 alone. This retrospective secondary data analysis study was approved by the University of Vermont Institutional Review Board.

### Study area and population

The University of Vermont Health Network (UVMHN) has locations throughout Vermont and upstate New York serving children and adolescents with asthma in outpatient settings. We obtained electronic health record (EHR) encounters (outpatient visits) for patients 3–21 years, diagnosed with asthma, living in Vermont and upstate New York, and seen in a UVMHN outpatient primary care office or pediatric pulmonary specialty clinic during 2022–2024.

### Outcome: asthma control

Encounters include data from an asthma “smart form” built in the EHR and used across the UVMHN’s pediatric and family medicine primary care and specialty clinics. The form allows clinicians to document medications used in asthma action plans and evaluate asthma control using three assessments: (1) the Test for Respiratory and Asthma Control in Kids (TRACK), (2) the Asthma Therapy Assessment Questionnaire (ATAQ), and (3) the National Heart, Lung, and Blood Institute (NHLBI) control charts. Each assessment evaluates both the impairment and risk domains of patients’ asthma control. These assessments are administered by office staff to the patient and their parent or guardian during patient intake.

#### TRACK

The TRACK, designed for children 0–4 years of age, uses five items assessed over the patient’s prior 4-week experience. These include the frequency of the child’s breathing problems, interference with normal activities, interference with sleep, treatment with rescue or quick-relief medications, and treatment with oral corticosteroids [29]. The TRACK asthma control score ranges from 0 to 100 with higher scores indicating better-controlled asthma. A score of less than 80 suggests that the patient has uncontrolled asthma [30].

#### ATAQ

The ATAQ for children and adolescents (ages 5 and older) includes seven yes/no questions about the patient’s symptoms, impacts to daily life, and patient sentiment about their asthma control level [31]. Like the TRACK, these are assessed over the prior 4 weeks. Responses to the questions are summed (with “yes” being a 1, and “no” being a 0), yielding an ATAQ asthma control score between 0 and 7. A higher score indicates worse control.

#### NHLBI guidelines

 The NHLBI guidelines are applicable to pediatric patients across the full age range and have five questions about patient symptoms and medication use over the prior 4 weeks. Patients are classified into three categories: well-controlled, not-well-controlled, and very-poorly-controlled. For our analysis, we grouped the not-well-controlled and very-poorly-controlled categories together as “not controlled,” because clinically, status in either one of these two categories would alert the provider that a change in therapy is needed.

### Canadian wildfire smoke PM_2.5_ sources

On June 1, 2023, lightning strikes caused over 120 wildfires in the Canadian province of Quebec [32]. Extreme drought conditions contributed both to the persistence and spread of these fires and to the ignition of new ones. In total, three peak wildfire periods occurred in Quebec in the summer of 2023, where burn area rates often were greater than 100,000 ha per day: June 1–12, June 19–28, and July 3–15 [32]. Weather patterns, including a low-pressure system over the Canadian Maritime Provinces and a high-pressure ridge to the west, directed wildfire smoke south and east into Upstate New York, Vermont, the broader eastern United States, and even across the Atlantic to Europe [33]. The nearest wildfire during our study site burned more than 300 km from the study area’s edge [32].

### Exposure: air quality

We evaluated smoke exposure using data compiled by the National Aeronautics and Space Administration (NASA) Health and Air Quality Applied Sciences Team (HAQAST), downloaded from the Hazardous Air Quality Ensemble System (HAQES) [34]. HAQES represents real-time total PM_2.5_ by integrating remote sensing and modeled data from various federal agencies including the National Oceanic and Atmospheric Administration, the Environmental Protection Agency, and NASA. We derived HAQES total surface PM_2.5_ at the census-tract level from data at a spatial resolution of 12 km. We did not compute a shared variance ($\:{R}^{2}$) statistic for the HAQES data; however, the HAQES model has been previously validated against ground-based monitor measurements, showing strong agreement between modeled and observed PM₂.₅ concentrations [35]. We then calculated daily averages for each census tract (based on 2020 census tract boundaries) from the mean of the eight 3-hour measurements for each day. No days were excluded as at least one 3-hour measurement was available for each day between May 1 and October 31 for 2022, 2023, and 2024. Over 90% of the daily averages were calculated with all eight 3-hour measurements. To match the spatial unit of our clinical encounters, we converted census-tract level daily averages to ZIP codes by using ArcGIS Pro (version 3.4.3). Using a spatial join with the intersect parameter, we calculated average daily PM_2.5_ from census-tracts that overlap in the same ZIP code polygon. Each overlapping census-tract within a ZIP code was weighted equally in the spatial join.

**Fig. 1:**
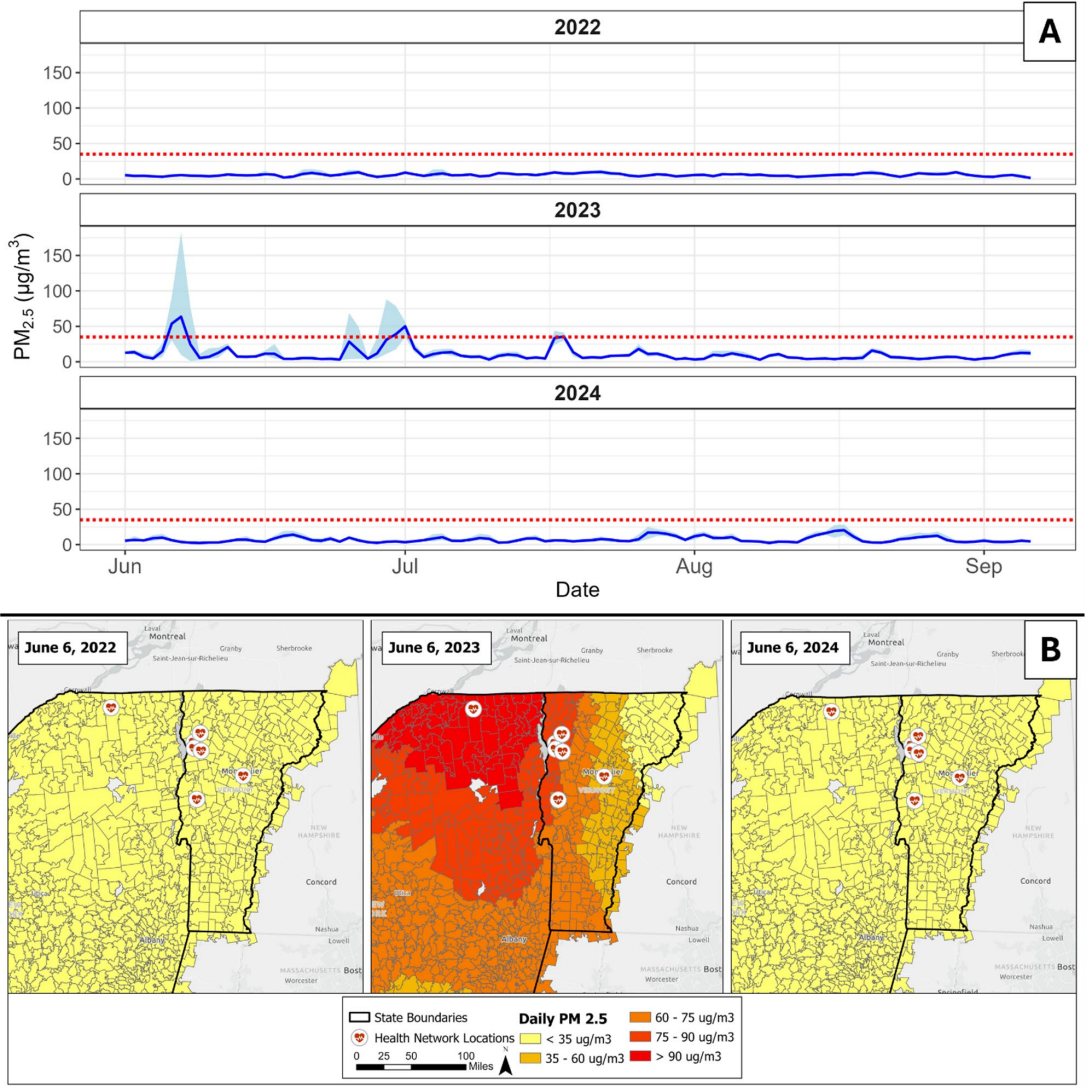
**A** Average (blue line) PM_2.5_ across the entire study area from June 1 – September 5. Note that our study period starts on June 6, but we have included additional days for reference. The shaded blue area falls within the minimum and maximum daily PM_2.5_. The dotted red line is at the threshold for harmful air quality, 35 µg/m^3^. **B** Average daily PM_2.5_ values for ZIP codes in Vermont and upstate New York on the first day of our study period (June 6) for years 2022, 2023, and 2024

We determined that June 6, 2023, was the first day that most ZIP codes in our study area exceeded the threshold for unhealthy air quality (Fig. 1). This threshold is defined by the US National Ambient Air Quality Standards as a 24-hour PM_2.5_ concentration greater than 35 µg/m^3^ [36]. We defined our sampling period as the 3-month (92-day) span after the first date (June 6 to September 5) to capture all PM_2.5_ peaks and potential delayed effects. The same time frame was used for 2022 and 2024. The categorical variable of year was then used as a proxy for wildfire smoke exposure, as the surrounding years did not experience unhealthy summer air quality on any day. During the 2023 sampling period, air quality rose to unhealthy levels on 6 days across our study area, reaching a maximum PM_2.5_ of 183 µg/m^3^ on June 7 in East Syracuse, NY.

**Fig. 2:**
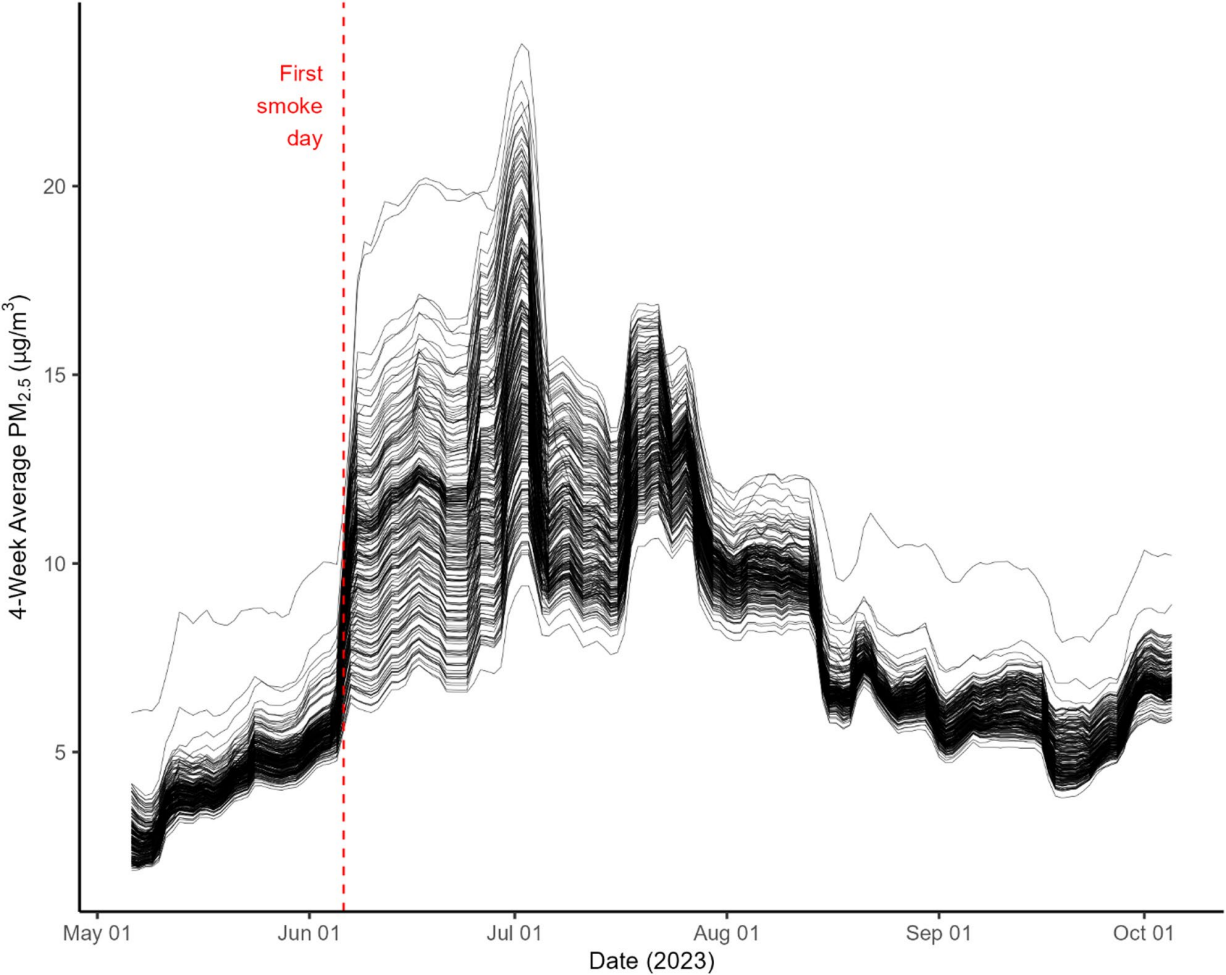
Airborne particulate matter over time for all 243 ZIP codes of patients seen during our study. Particulate matter is measured as 4-week average PM_2.5_ concentrations. Our study period is June 6 – September 5, 2023, but data is presented from May 6 – October 5, 2023 for context. Each black line represents a single ZIP code. The dashed red line is the start date of our sampling period, June 6

For our second analysis, we calculated the 4-week rolling average PM_2.5_ for each ZIP code and each day of the wildfire summer (2023) using the same ZIP code–level daily PM_2.5_ averages described above (Fig. 2). These rolling averages matched the 4-week period patients were asked to consider in the three clinical asthma control assessments. For descriptive purposes, we also calculated the daily range of the 4-week rolling average PM₂.₅ across all ZIP codes, which allowed us to quantify the extent of spatial variation in exposure during the summer.

### Covariates

Airborne pollen, which can vary in intensity year to year, is known to be a trigger for school-aged children with asthma [37, 38]. To control for inter-annual differences in pollen exposure, we obtained daily total pollen counts for Vermont from the Vermont Open Geodata Portal for 2022, 2023, and 2024 [39]. Because pollen counts were not readily available for New York, and because these counts were given for the full state of Vermont rather than smaller regions or ZIP codes, we treated pollen as a broadened binary variable. Encounters in a month containing at least one total pollen count in the “very high” threshold range (greater than 1,500 for tree pollen) were considered high pollen exposure periods [40], while all other pollen levels were considered low pollen exposure periods.

Extreme temperatures and humidity as well as sudden changes in temperature and humidity are also known to be asthma triggers [41, 42]. We evaluated daily temperature and humidity as potential covariates. Because mean values did not differ significantly among study years, they were not included in the final analyses.

### Statistical analysis

For the first analysis, we compared asthma control between the smoke-affected summer months of 2023 (June 6 – Sept. 5) and the same summer months of unaffected years (2022 and 2024). To assess demographic differences between years, we used t-tests for age, and chi-squared tests for gender, race, and state of residence. Participants with missing or unreported race (*n* = 23) or gender (*n* = 1) were excluded from the respective comparisons. To ensure an independent sample, we retained the encounter with the worst asthma control level for patients seen more than once in a summer. For our continuous variables of TRACK and ATAQ scores, we used multiple linear regression models to assess the relationship between mean asthma control score and year (2022, 2023, 2024), controlling for high pollen exposure. The year 2023 was treated as the reference year, as it corresponded to the period affected by wildfire smoke. Results are reported as mean differences in asthma control score and 95% confidence intervals (CI). For our binary outcome variable of controlled/uncontrolled asthma measured by the NHLBI guidelines, we used a multiple logistic regression model to assess the odds of uncontrolled asthma across years (2022, 2023, 2024), again controlling for periods of high pollen levels. Results are reported as odds ratios of uncontrolled asthma with 95% CI.

For the second analysis, which focused only on the smoke-affected year of 2023, we assessed the relationship between asthma control and 4-week average PM_2.5_ across the ZIP codes in our study area. For the continuous variables of TRACK and ATAQ, we used simple linear regression models of asthma control versus 4-week average PM_2.5_. Results are reported as mean differences in asthma control and 95% CI. For the binary outcome variable of the NHLBI guidelines (controlled/uncontrolled), we used a simple logistic regression model to evaluate the association between the odds of uncontrolled asthma and 4-week average PM_2.5_. These results are reported as odds ratios of uncontrolled asthma with 95% CI.

## Results

The demographic distribution of our sample across the three years appears in Table 1. Our analyses included 1,217 unique encounters, with some patients contributing one encounter per year across multiple years. Across all encounters, most were for patients identified as white (81% of those with reported race) and from Vermont (77%). The average age of patients was 3.5 years (SD = 0.5, *n* = 172) for TRACK, 9.7 years (SD = 3.7, *n* = 923) for ATAQ, and 9.0 years (SD = 4.5, *n* = 995) for NHLBI. Most encounters (75%) occurred at a single pediatric pulmonology specialty clinic in Vermont, which sees patients from across the study area due to its specialized nature.

**Table 1:** Patient characteristics of each year, including patients evaluated by any of the three asthma control assessments (TRACK, ATAQ, and NHLBI guidelines)

Variables	2022 *n* = 324	2023 *n* = 421	2024 *n* = 472
Age group			
3–4 years	56 (17.3%)	75 (17.8%)	55 (11.7%)
5–21 years	268 (82.7%)	346 (82.2%)	417 (88.3%)
Gender			
Male	183 (56.5%)	247 (58.7%)	260 (55.1%)
Female	141 (43.5%)	174 (41.33%)	211 (44.7%)
Missing	0	0	1 (0.2%)
Race			
White	269 (83.0%)	342 (81.2%)	377 (79.9%)
Non-white	51 (15.7%)	74 (17.6%)	81 (17.2%)
Missing or declined to respond	4 (1.2%)	5 (1.2%)	14 (3.0%)
State of Residence			
New York	66 (20.4%)	101 (24.0%)	114 (24.2%)
Vermont	258 (79.6%)	320 (76.0%)	358 (75.9%)

*TRACK* Test for Respiratory and Asthma Control in Kids,* ATAQ* Asthma Therapy Assessment Questionnaire, *NHLBI* National Heart, Lung, and Blood Institute

**Fig. 3:**
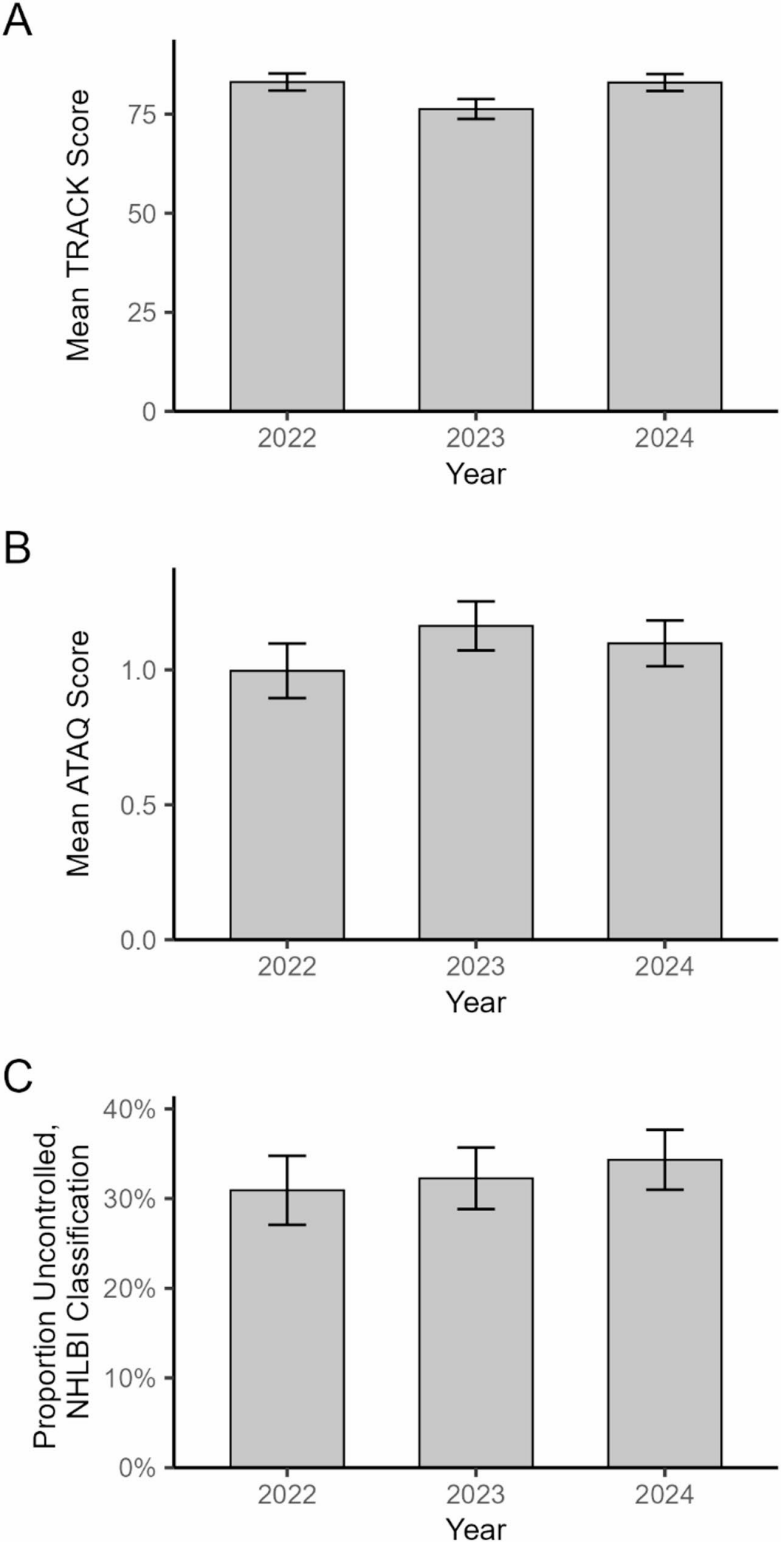
Comparison of mean asthma control between a smoke-affected year (2023) and two non-smoke-affected years (2022 and 2024). **A** TRACK: higher scores signify better control. **B** ATAQ: higher scores signify worse control. **C **Proportion of patients with uncontrolled asthma, as measured using the NHLBI guidelines. Annual means are across all patients seen between June 6 and September 5. See Tables 2, 3 and 4 for corresponding statistical results

When comparing asthma control across years, we found evidence of an association between asthma control and wildfire smoke exposure, though results differed across the three clinical asthma control measures. For TRACK scores (in which a lower score corresponds to worse control), we found a nearly 7-point lower TRACK mean score in 2023 than in 2022 (*p* = 0.048), and a similar but not statistically significant difference between 2023 and 2024 (*p* = 0.054) (Fig. 3A; Table 2). The ATAQ mean scores (in which higher scores correspond to worse control) in 2023 were nearly 0.4 points lower than in 2022 (*p* = 0.020), and scores showed a similar but not statistically significant difference from 2023 to 2024 (*p* = 0.067) (Fig. 3B; Table 3). For our third asthma control assessment based on the NHLBI guidelines, there were no differences in the predicted odds of uncontrolled asthma comparing 2023 to 2022 (*p* = 0.455) or 2023 to 2024 (*p* = 0.992) (Fig. 3C; Table 4). Of note, high pollen exposure was associated with worse asthma control in the ATAQ (*p* < 0.001), but not in the other two assessments.

**Table 2:** Multiple linear regression results for asthma control score, as measured by the TRACK assessment, associated with year and level of pollen (Analysis 1)

Variable	Coefficient (β)	SE	*p*-value	95% CI (low)	95% CI (high)
Year (June 6 – September 5)					
2023 (Smoke-affected)	REF				
2022	6.80*	3.41	0.048	0.07	13.53
2024	6.66	3.43	0.054	-0.11	13.44
Pollen					
Low	REF				
High	0.11	4.18	0.979	-8.15	8.37

*TRACK* Test for Respiratory and Asthma Control in Kids, *SE* Standard Error,* CI* Confidence Interval

*Indicates statistical significance at *p* < 0.05

**Table 3:** Multiple linear regression results for asthma control score, as measured by the ATAQ assessment, associated with year and level of pollen (Analysis 1)

Variable	Coefficient (β)	SE	*p*-value	95% CI (low)	95% CI (high)
Year (June 6 – September 5)					
2023 (Smoke-affected)	REF				
2022	-0.34*	0.14	0.020	-0.62	-0.05
2024	-0.24	0.13	0.067	-0.50	0.02
Pollen					
Low	REF				
High	0.54*	0.14	< 0.001	0.27	0.81

*ATAQ* Asthma Therapy Assessment Questionnaire,* SE* Standard Error, *CI* Confidence Interval

*Indicates statistical significance at *p* < 0.05; p-values < 0.001 are reported as *p* < 0.001

**Table 4:** Logistic regression results for the odds ratio of uncontrolled asthma, as measured by the NHLBI guidelines, associated with year and level of pollen (Analysis 1)

Variable	Odds Ratio	SE	*p*-value	95% CI (low)	95% CI (high)
Year (June 6 – September 5)					
2023 (Smoke-affected)	REF				
2022	0.87	0.16	0.455	0.60	1.25
2024	1.00	0.17	0.992	0.72	1.39
Pollen					
Low	REF				
High	1.32	0.23	0.120	0.93	1.87

*NHLBI* National Heart, Lung, and Blood Institute, *SE* Standard Error, *CI *Confidence Interval

**Fig. 4:**
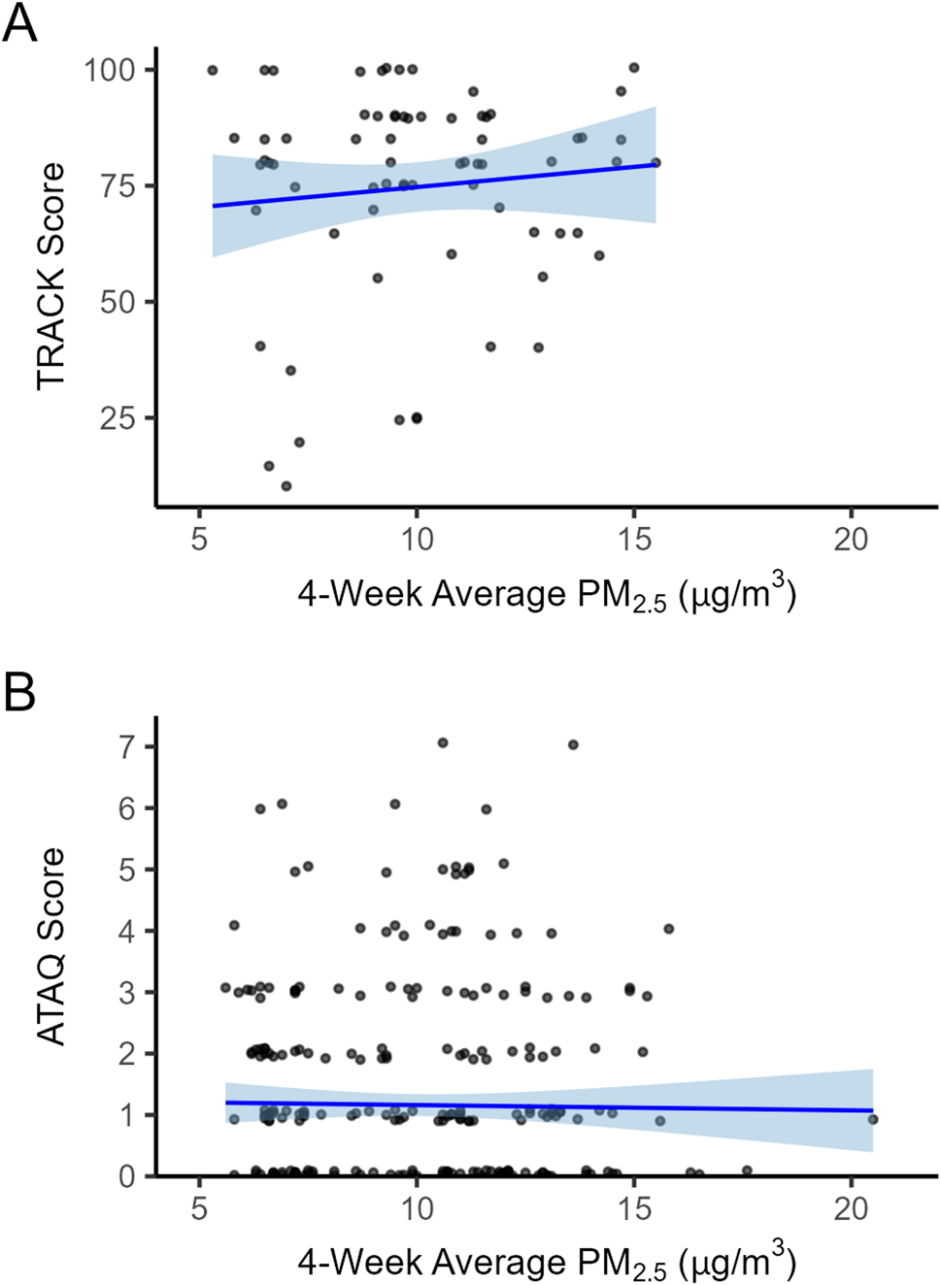
Relationships between asthma control and airborne particulate matter among ZIP codes in the smoke-affected year of 2023. **A** TRACK: higher values signify better control. **B** ATAQ: higher values signify worse control. Both panels: particulate matter measured as 4-week average PM_2.5_. Blue lines and shading indicate simple linear regressions and 95% confidence intervals, respectively. Individual data points are jittered slightly along the y-axis to improve visibility of overlapping values

In our analysis within the severely smoke-affected summer of 2023, we observed no significant spatial associations between particulate matter and any of the three clinical measures of asthma control. TRACK scores (Figure 4A) did not worsen with increased PM_2.5_ (β = 1.09, 95% CI: -0.95, 3.13, *p* = 0.290). ATAQ scores (Figure 4B) were not different as PM_2.5_ increased (β = -0.01, 95% CI: -0.07, 0.05, *p*= 0.801). Finally, the odds of uncontrolled asthma using the NHLBI guideline charts were not associated with higher PM_2.5_ (β = 0.99, 95% CI: 0.91, 1.07, *p* = 0.716). Across the summer, the daily range in 4-week rolling average PM₂.₅ across ZIP codes spanned 5.8–18.0 µg/m³, with the smallest variation on August 20 and the largest on June 29 (Figure 2), indicating that while all ZIP codes experienced distinct PM₂.₅ levels, the magnitude of differences varied from day to day.

## Discussion

This study is the first to investigate the effect of wildfire smoke on pediatric asthma in the Northeastern US. Our findings suggest a more nuanced relationship than expected. Two of our three asthma control assessments indicated significantly negative impacts of wildfire smoke in our year-to-year assessment, but we found no association in the spatial analysis using ZIP code–level PM_2.5_ exposure. Our study affirms the need to consider wildfire smoke in asthma management, even in regions far from fires. It also underscores the need for more research to fully understand this exposure-response relationship.

The exact mechanism of PM_2.5_ from wildfire smoke to induce asthma exacerbations is unclear but is an active area of inquiry. Tuazon identified three general mechanisms by which air pollution could contribute to the development of asthma: genetic factors, altered gene expression, and microbiome changes [43]. Acute inflammation, oxidative stress, airway epithelial injury, and epigenetic changes were found to be likely mechanisms for the development of asthma in response to wildfire smoke [44]. Inflammatory cytokines have been identified in animal models and a limited number of human trials, though the exact mechanism is not fully understood [45]. Increased incidence and mortality from Covid-19 associated with wildfire smoke and PM_2.5_ suggest other possible mechanisms related to altered lung defenses and immune function [46]. Taken together, these findings provide a range of mechanistic rationale for our examination of the influence of wildfire smoke exposure on asthma control.

 ID="Par508">Prior studies have demonstrated strong and consistent relationships between wildfire smoke and negative respiratory health outcomes [21], and that children are a particularly vulnerable population [19, 20]. In contrast, we find more nuanced results. One reason for this may be that Our finding that children’s asthma previous studies have largely used emergency department (ED) visits and hospitalizations as their outcome variable. In fact, in the most recent meta-analysis of the effects of wildfire smoke on children’s health, all 39 of the included studies relating to respiratory mortalities and morbidities used either ED visits, hospitalizations, or clinic visits [20]. Our approach was distinct in its use of three outpatient measures of asthma control. These questionnaires can capture the full spectrum of asthma control, including minor impairments not associated with ED visits [47]. Future studies could compare acute and non-acute asthma outcomes within the same populations and wildfire smoke events to better understand how different outcome types capture health impacts of smoke exposure.

Another contributor to our nuanced results may be the distance from fires and density of smoke in our study. Previous studies have focused mostly on health impacts to populations in close geographic proximity to a wildfire, while the closest wildfire to our study area was more than 300 km away [32]. The resulting poor air quality in Vermont and upstate New York occurred as isolated “spikes” over a day or two at a time, at comparatively lower levels. The highest PM_2.5_ value estimated in our study area was less than 200 µg/m^3^. Other studies that have found clear relationships between smoke and health report much greater PM_2.5_ values of 295 µg/m^3^ [48], greater than 400 µg/m^3^ [49], and greater than 5000 µg/m^3^ [50]. Studies also report extended periods of consistent smoke, such as the sustained month-long episode of hazardous air quality caused by California wildfires in 2008 [51] and the “summer of smoke” in Northwest Territories, Canada in 2014 [52]. In one study of distant wildfire smoke, hospitalization rates for elderly populations in US Atlantic states increased with exposure to Canadian wildfire smoke, but this study reported a maximum PM_2.5_ value of 338 µg/m^3^ [53], which is more than 80% higher than the maximum PM_2.5_ observed in our study. Researchers in Australia observed results more comparable to ours, finding no significant differences in several respiratory health outcomes when patients were exposed to lower levels (max PM_2.5_ of 36.5 µg/m^3^) of smoke [54]. Future studies could investigate if a threshold exists in wildfire smoke density and duration that measurably impacts certain respiratory health outcomes.

Our finding that children’s asthma control is worse in a summer with wildfire smoke is particularly important for clinical decision-making. Understanding seasonal patterns has historically guided asthma therapy [55]. Summer is a time when providers anticipate better asthma outcomes and may consider stepping down controller medications [56]. The risk of summer wildfire smoke complicates this decision. As climate change extends wildfire [13] and pollen [57] seasons, these environmental triggers may increasingly overlap, intensifying seasonal exacerbations and challenging previously reliable trends. Patient education about protective behaviors during wildfire smoke, when integrated into routine care, has been linked to improved outcomes [58]. Our study emphasizes the importance of providing anticipatory guidance about restarting asthma controller medications, using rescue medications, and limiting exposure to poor air quality.

There are several study limitations worth noting. First, our data were drawn from a single health network and may not represent the entire pediatric population of Vermont and upstate New York. However, the UVMHN is the largest regional health system and the only tertiary care center in the area. Second, utilizing the asthma smart form, which produced our outcome variables, encourages a unique level of proactive clinical care that may have contributed to better than expected health outcomes. Our results may not be generalizable to health networks that do not use such a tool. Third, utilizing a patient’s home ZIP code to assign smoke exposure may not capture their true experience. Patients often spend time away from home in summer and may have even traveled out of our study region but returned for a scheduled office visit. Fourth, our use of a single pollutant (PM_2.5_) as the predictor variable does not capture the whole range of harmful pollutants present in wildfire smoke, including ozone, nitrogen oxides, and other particulates. We selected PM_2.5_ because it is the most widely used and reported wildfire-related exposure metric in the literature to date [20]. Future research should investigate the health impacts of additional wildfire-related air pollutants. Finally, acute responses during a wildfire smoke event such as telephone calls to the specialty clinic, prescription of steroids, and ED visits were not examined in this study and should be investigated in future work.

## Conclusions

In this study, we found associations between episodic summertime wildfire smoke exposure and worsened asthma control across years, but not for all assessment tools. We observed no associations between asthma control and ZIP code–level PM_2.5_concentrations within the severely smoke-affected summer of 2023. These findings suggest that while wildfire smoke may negatively affect asthma control, the impact may not be fully captured by ambient PM_2.5_ levels nor consistently detected across different clinical assessment tools. As wildfire seasons lengthen and intensify, diverse assessment tools, interdisciplinary research, and close collaboration with healthcare providers will be essential to protect vulnerable pediatric populations.

## Data Availability

The datasets analyzed in the current study are not publicly available due to patient privacy concerns and restrictions outlined in the Institutional Review Board’s approval of our study. A simulated dataset and the Stata code used for this analysis are available from Figshare.

## Abbreviations

ATAQ: Asthma Therapy Assessment Questionnaire
CI: Confidence Interval
ED: Emergency Department
EHR: Electronic Health Record
HAQAST: Health and Air Quality Applied Sciences Team
HAQES: Hazardous Air Quality Ensemble System
NASA: National Aeronautics and Space Administration
NHLBI: National Heart, Lung and Blood Institute
SD: Standard Deviation
SE: Standard Error
TRACK: Test for Respiratory and Asthma Control in Kids
US: United States
UVMHN: University of Vermont Health Network
WHO: World Health Organization
